# Enhanced and Enduring Protection against Tuberculosis by Recombinant BCG-Ag85C and Its Association with Modulation of Cytokine Profile in Lung

**DOI:** 10.1371/journal.pone.0003869

**Published:** 2008-12-04

**Authors:** Ruchi Jain, Bappaditya Dey, Neeraj Dhar, Vivek Rao, Ramandeep Singh, Umesh D. Gupta, V. M. Katoch, V. D. Ramanathan, Anil K. Tyagi

**Affiliations:** 1 Department of Biochemistry, University of Delhi South Campus, New Delhi, India; 2 National JALMA Institute for Leprosy & Other Mycobacterial Diseases, Tajganj, Agra, India; 3 Department of Clinical Pathology, Tuberculosis Research Center, Chetpet, Chennai, India; 4 Laboratory of Bacteriology, Global Health Institute, Ecole Polytechnique Federale de Lausanne, Lausanne, Switzerland; 5 Division of mycobacterial research, The National Institute for Medical Research, The Ridgeweay Mill Hill, London, United Kingdom; 6 Tuberculosis Research Section, Laboratory of Clinical Infectious Diseases, National Institute of Allergy and Infectious Diseases, National Institutes of Health, Bethesda, Maryland, United States of America; Universita di Sassari, Italy

## Abstract

**Background:**

The variable efficacy (0–80%) of *Mycobacterium bovis* Bacille Calmette Guréin (BCG) vaccine against adult tuberculosis (TB) necessitates development of alternative vaccine candidates. Development of recombinant BCG (rBCG) over-expressing promising immunodominant antigens of *M. tuberculosis* represents one of the potential approaches for the development of vaccines against TB.

**Methods/Principal Findings:**

A recombinant strain of BCG - rBCG85C, over expressing the antigen 85C, a secretory immuno-dominant protein of *M. tuberculosis*, was evaluated for its protective efficacy in guinea pigs against *M. tuberculosis* challenge by aerosol route. Immunization with rBCG85C resulted in a substantial reduction in the lung (1.87 log_10_, *p*<0.01) and spleen (2.36 log_10_, *p*<0.001) bacillary load with a commensurate reduction in pathological damage, when compared to the animals immunized with the parent BCG strain at 10 weeks post-infection. rBCG85C continued to provide superior protection over BCG even when post-challenge period was prolonged to 16 weeks. The cytokine profile of pulmonary granulomas revealed that the superior protection imparted by rBCG85C was associated with the reduced levels of pro-inflammatory cytokines - interleukin (IL)-12, interferon (IFN)-γ, tumor necrosis factor (TNF)-α, moderate levels of anti-inflammatory cytokine - transforming growth factor (TGF)-β along with up-regulation of inducible nitric oxide synthase (iNOS). In addition, the rBCG85C vaccine induced modulation of the cytokine levels was found to be associated with reduced fibrosis and antigen load accompanied by the restoration of normal lung architecture.

**Conclusions/Significance:**

These results clearly indicate the superiority of rBCG85C over BCG as a promising prophylactic vaccine against TB. The enduring protection observed in this study gives enough reason to postulate that if an open-ended study is carried out with low dose of infection, rBCG85C vaccine in all likelihood would show enhanced survival of guinea pigs.

## Introduction

*Mycobacterium tuberculosis* continues to be a leading cause of human deaths due to an infectious agent [1]. The situation has become even more precarious due to the emergence of multi drug resistant strains of *M. tuberculosis* and lethal combination of tuberculosis (TB) and HIV infections [2], [3]. It has been indisputably accepted by TB experts that complete eradication of this disease may be difficult to achieve without the availability of an efficient vaccine. *Mycobacterium bovis* Bacille Calmette Guréin (BCG), the only vaccine currently in use against TB, despite its satisfactory performance against childhood TB, does not impart adequate protection against pulmonary TB in adults, with its efficacy ranging from 0–80% [4], [5], [6].

Development of recombinant BCG (rBCG) based vaccines over-expressing promising immuno-dominant antigens of *M. tuberculosis* represents one of the potential approaches to improve upon the performance of BCG [7], [8], [9], [10]. The proteins belonging to the antigen 85 (Ag85) complex, a family of 30–32 kDa proteins (Ag85A, Ag85B and Ag85C) represent a group of the major secretory and immunodominant proteins of *M. bovis* BCG and *M. tuberculosis* [11], [12] leading to their inclusion in several approaches for the development of vaccines against TB [13], [14], [15], [16]. Of the three members of the Ag85 complex, Ag85C (*fbpC*, *Rv0129c*), in particular, significantly contributes towards the mycolyl transferase activity of *M. tuberculosis* and is singularly responsible for almost 40% mycolate content of this pathogen [17], [18]. The mycolyl transferase activity specific to Ag85C cannot be substituted by the other two members i.e. Ag85A and 85B as shown by the reduction in the mycolic acid content in the mutant of *M. tuberculosis* lacking Ag85C activity [18], [19]. In addition to its role in cell wall biosynthesis, it has also been shown to be highly immuno-dominant in nature, with several epitopes recognized by CD4 and CD8 T cells [20], [21], [22]. In addition, a preferential recognition of Ag85C over the other two members of the Ag85 complex, by sera obtained from childhood TB patients especially by the smear and culture negative patients, further signifies its immunodominant nature [23]. Moreover, gene encoding Ag85C is known to be up-regulated in the activated macrophages infected with *M. tuberculosis*, perhaps allowing the bacilli to thicken its cell wall in order to resist the onslaught of the bactericidal mechanisms of the host [24]. The extra-cellular abundance of Ag85C and its immunodominant nature makes this antigen an attractive target for the development of anti-TB vaccines. We have earlier reported the construction of rBCG strains over-expressing the immuno-dominant antigens of Ag85 complex under the transcriptional control of mycobacterial promoters of varying strength [25]. The immunogenicity of some of these rBCG strains was also studied in murine model [14], [26]. In the present study, the protective efficacy of rBCG85C was assessed in a highly susceptible guinea pig model along with the evaluation of immune responses following *M. tuberculosis* challenge by the aerosol route. Immunization of guinea pigs with rBCG85C resulted in a significantly enhanced protection characterized by a marked reduction in bacillary load in lungs and spleen along with a significantly reduced pathology in various organs, when compared to BCG immunization, at least up to 16 weeks post-infection.

Furthermore, in order to gain an insight into the immunological basis of protection and disease-associated pathology, expression of cytokines and presence of mycobacterial antigens were measured in pulmonary granulomatous lesions by immunohistochemistry (IHC). In addition, semi-quantitative real time PCR (qPCR) for various cytokines (IL-12, IFN-γ, TNF-α and TGF-β) and inducible nitric oxide synthase (iNOS) was also performed on the total RNA isolated from lung tissues. Analysis of various immuno-pathological parameters that influence protective efficacy demonstrated that rBCG85C vaccine induced modulation of the cytokine levels was associated with the reduced bacillary and antigen load accompanied by the restoration of normal lung architecture.

## Materials and Methods

### Bacteria

*M. bovis* BCG (Danish strain) was procured from BCG laboratories, Chennai, India. *M. tuberculosis* H37Rv was kindly provided by Dr. J. S. Tyagi, All India institute of medical sciences, New Delhi, India. BCG, rBCG85C and *M. tuberculosis* strains were grown to mid-log phase in Middle Brook (MB) 7H9 media and stocks were prepared as described earlier [27].

### Preparation of antigens for immunization

For preparation of rBCG85C, a *Mycobacteria - Escherichia coli* shuttle plasmid pSD5.pro was used as described earlier [25], [28]. Briefly, the plasmid (pSD5.pro) was engineered to over-express Ag85C along with its native signal sequence under transcriptional control of the promoter of *M. leprae* gene encoding heat shock protein 65 (*hsp65*). The plasmid was electroporated into *M. bovis* BCG and selected on MB7H11 plates containing Kanamycin (25 µg/ml).

### Experimental animals

Pathogen free 200–300 g female outbred guinea pigs (Dunkin Hartley strain) used for the protective efficacy studies were procured from Disease Free Small Animal House Facility, Haryana Agricultural University, Hissar, India. The animals were housed in stainless steel cages and were provided with *ad libitum* food and water in a BSLIII facility (National JALMA Institute of Leprosy and Other Mycobacterial Diseases, Agra, India). All the experimental protocols were reviewed and approved by the animal ethics committee of the institute.

### Immunization and aerosol challenge of guinea pigs with *M. tuberculosis*

For evaluation of protective efficacy, two experiments were carried out by varying the interval between (i) immunization and infection and (ii) infection and euthanasia. In each experiment, guinea pigs (n = 6) were immunized with 5×10^5^ CFU of either BCG (Danish strain) or rBCG85C in 100 µl of saline by intra-dermal (i.d.) route. In the control group, guinea pigs were injected with 100 µl of saline (i.d.).

In Exp-I, guinea pigs were challenged 6 weeks post immunization with ∼500 bacilli of virulent *M. tuberculosis* H37Rv *via* the respiratory route in an aerosol chamber (Inhalation exposure system, Glasscol Inc., IN, USA) and were euthanized 10 weeks following the infection. In Exp-II, the time interval between immunization and challenge was extended to 12 weeks and animals were euthanized 16 weeks post infection.

### Measurement of protective efficacy

Animals were monitored regularly for change in body weight and general body condition as an indicator of disease progression and were euthanized at specified time points. In addition to the measurement of bacillary load in lung and spleen, gross and histopathological changes in various organs and extent of pulmonary fibrosis were evaluated. A significant reduction in these parameters in vaccinated animals was considered as a protective effect of the vaccine.

### Necropsy procedure and gross pathological evaluation

Guinea pigs were euthanized by i.p. injection of Thiopentone sodium (100 mg/kg body weight) (Neon Laboratories Ltd., India). After aseptically dissecting the animals, lung, liver and spleen were examined for gross pathological changes and scored using the Mitchison scoring system [29] with minor modifications (Table S1), wherein equal emphasis was given to each organ. For histopathological evaluation, three lung lobes (right caudal, middle and cranial) and a portion of left dorsal lobe of liver were removed and fixed in 10% neutral buffered formalin. Left caudal lung lobe and cranial portion of spleen were aseptically removed for the measurement of bacillary load. A portion of left cranial lung lobe and caudal portion of spleen were stored in RNA *later®* (Ambion, TX, USA) at −20°C for isolation of RNA to be used for real time RT-PCR studies.

### Bacterial enumeration

Specific portions of lungs and spleen were weighed and homogenized separately in 5 ml saline in a Teflon glass homogenizer. Appropriate dilutions of the homogenates were inoculated on to MB7H11 agar plates in duplicates and incubated at 37°C in a CO_2_ incubator for three to four weeks. The number of colonies were counted and expressed as log_10_ CFU/g of tissue. The detection limit in case of both lung and spleen CFU was 1.0 log_10_ CFU/g.

### Histopathological evaluation

Sections of 5 µm thickness from formalin fixed and paraffin embedded tissues were cut on to glass slides and stained with haematoxylin and eosin for histo-pathological examination. The percent granuloma in lung and liver, type and extent of necrosis, organization of granuloma along with the type of infiltrating cells were assessed as described earlier [30]. In order to determine the extent of collagen deposition and fibrosis, the lung sections were also stained with Van Gieson stain.

### Immunohistochemistry

Deparaffinized and re-hydrated lung sections were quenched for endogenous peroxidase with 3% hydrogen peroxide (in methanol) followed by antigen retrieval at 90–100°C for 10 min in citrate buffer (pH-6.5). After blocking the non-specific sites with 2% BSA and 4% goat sera in PBS, sections were probed with rabbit polyclonal anti-sera against guinea pig IFN-γ, TNF-α (kindly provided by Dr. DN McMurray, The Texas A&M University System Health Science Center, TX, USA) and Ag85 complex of *M. tuberculosis* (raised in our laboratory) overnight at 4°C. Following washing with PBST (containing 0.1% Triton-×100 and 0.5% BSA) and PBS three times, sections were treated with horseradish peroxidase (HRP) conjugated goat anti-rabbit antiserum (Jackson laboratories, PA, USA). Finally, the antibody bound antigenic sites were detected by a colored reaction (brown) using diaminobenzedine as a chromogenic substrate for HRP and the slides were counterstained with Mayer's haematoxylin. Negative controls were treated with similar procedure except that primary antibody was replaced with normal rabbit sera.

### Image analysis

The tissue sections were examined by light microscopy and the images were captured by using a CCD camera DS-Fi1-U2 (Nikon Corp., Tokyo, Japan). The whole section was examined to determine area and intensity of staining. Immuno-reactivity was manually scored by estimating the area showing characteristic staining (A, 1 = <10%, 2 = 10–25%, 3 = 25–50%, 4 = >50%) and by estimating the intensity of staining (I, 1 = weak, 2 = moderate, 3 = strong, 4 = very strong). A quick score (Q) was calculated for each slide by the formula (Q = A×I). The quick score values were categorized as low (1–2), moderate (3–6) and high (8–16).

### RNA extraction and real time RT-PCR

Total RNA was isolated from lung tissues using RNeasy mini columns and contaminating genomic DNA was removed by on column treatment with RNase free DNase (Qiagen Inc, CA, USA). Approximately 3 µg of total RNA from each animal was reverse transcribed by using random hexamers and Omniscript RT kit (Qiagen Inc, CA, USA) as per the manufacturer's instructions. Primers for guinea pig IFN-γ, TNF-α, TGF-β and IL-12 were designed with Primer Express software (Applied Biosystems, CA, USA) by using cDNA sequences available in the public database (http://www. ncbi.nlm.nih.gov) (Table S2). Primers for iNOS and 18S rRNA were used as described earlier [31], [32]. Real time PCR was performed by using SYBR green PCR Master Mix (Applied Biosystems, CA, USA) as per the manufacturer's instructions.

### Statistical analysis

Mean differences for Log_10_ CFU and % fold induction in mRNA expression levels as measured by real time PCR were analyzed by one-way analysis of variance (ANOVA). Least square difference and Duncan's post hoc tests were also carried out to determine the significance of differences between various groups. The differences between scores allotted for gross pathological lesions, granuloma percent, quick score (Q) for IHC and extent of collagen deposition across different groups were analysed by non-parametric methods. The non-parametric Kruskal-Wallis test was employed for comparison of multiple groups, followed by the Mann-Whitney *U* test for comparison between two groups. The differences were considered statistically significant when the *p* values were less than 0.05. These statistical tests were run on SPSS software (Version. 10.0, SPSS Inc., Illinois, USA).

## Results

### rBCG85C vaccination limits bacillary multiplication

**Figure 1:**
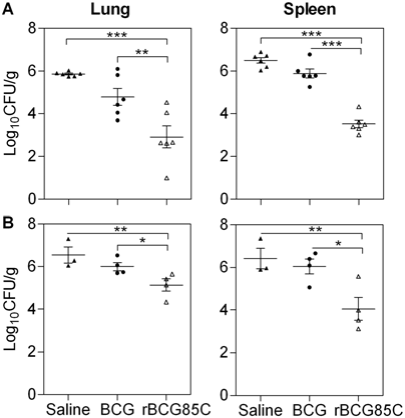
Superior protection by rBCG85C vaccination against *M. tuberculosis* challenge. The figure depicts the bacillary load in the lungs and spleen of immunized and saline treated guinea pigs (n = 6) at (A) 10 weeks (Exp-I) and (B) 16 weeks (Exp-II) post-infection. Immunization with rBCG85C resulted in a significantly lower bacillary load in lung and spleen, when compared to both BCG and saline groups. Each point represents the Log_10_ CFU value for an individual animal and the bar depicts mean (±SE) for each group. The lower limit of detection was 1.0 log_10_ CFU/g of tissue and animals with undetectable bacilli were allotted a CFU value of 1.0 log_10_/g. Missing data points represent the animals that succumbed to disease before the time of euthanasia. *, *p*<0.05; **, *p*<0.01 and ***, *p*<0.001 (One way ANOVA).

To evaluate the efficacy of rBCG85C vaccination, following an aerosol challenge with *M. tuberculosis*, bacillary load in the lungs and spleen of guinea pigs was determined. In experiment (Exp)-I, guinea pigs were infected with *M. tuberculosis* 6 weeks post-immunization and were euthanized at 10 weeks post-infection. Immunization with both BCG as well as with rBCG85C resulted in a marked reduction in the lung and spleen bacillary load, when compared to the saline treatment (Fig. 1A). However, the extent of reduction in case of rBCG85C immunized guinea pigs was significantly greater, when compared to BCG immunized animals (by 1.87 log_10_ in lung, *p*<0.01 and by 2.36 log_10_ in spleen, *p*<0.001). In a subsequent study (Exp-II), wherein guinea pigs were infected 12 weeks post-immunization and euthanized at 16 weeks post-infection, BCG vaccination exhibited a considerable decline in its ability to impede bacillary multiplication, as was evident from a comparable bacillary count in BCG and saline treated animals. However, bacillary load in rBCG85C-immunized animals was significantly lower, when compared to BCG immunized animals (by 0.87 log_10_ in lung, *p*<0.05 and by 1.99 log_10_ in spleen, *p*<0.05) (Fig. 1B). These observations clearly indicated enhanced protective efficacy of rBCG85C, when compared to BCG.

### Influence of rBCG85C vaccination on gross pathology

**Figure 2:**
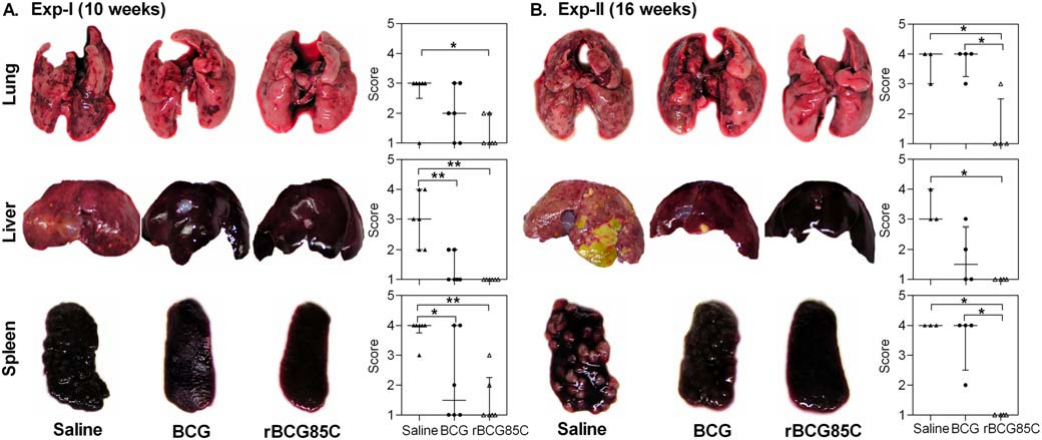
Reduction in gross pathological lesions in rBCG85C vaccinated animals following *M. tuberculosis* infection. The figure depicts representative photographs of lungs, liver and spleen of vaccinated and saline treated animals (n = 6) euthanized at (A) 10 weeks (Exp-I) and (B) 16 weeks (Exp-II) post-infection. Based on the extent of involvement, number and size of tubercles, areas of inflammation and necrosis, gross pathological scores were graded from 1–4 (Table S1) and represented graphically. Each point represents score for an individual animal and the bar depicts median (±inter quartile range) for each group. Missing data points represent the animals that succumbed to disease before the time of euthanasia. Immunization with rBCG85C resulted in fewer and smaller pulmonary, hepatic and splenic lesions when compared to both BCG and saline group. *, *p*<0.05 and **, *p*<0.01 (Mann-Whitney *U* test).

The trend in lung and spleen bacillary load was also substantiated by the gross pathological changes. At 10 weeks post-infection (Exp-I), severe pathological damage was observed in case of saline treated animals characterized by extensive involvement and numerous large tubercles with scattered areas of necrosis in both lung and liver (Fig. 2A). In addition, a marked enlargement of spleen with numerous large and small sized tubercles with occasional attrition of capsular structure was also observed in most of the saline treated animals. Guinea pigs immunized with BCG or rBCG85C showed a significant reduction in gross pathological damage, when compared to the saline treated animals. However, lesions were predominantly scanty and extremely small in the organs of rBCG85C-immunized animals, when compared to the BCG immunized animals (*p*<0.01) (Fig. 2A). At 16 weeks post-infection (Exp-II), 50% of the saline treated animals succumbed to the disease (3/6); animals that survived showed characteristic signs of end stage TB with extensive pathological damage (Fig. 2B). Although, the number of animals that survived until the time of euthanasia was similar in both the vaccinated groups (4/6), surviving animals in the BCG group showed extensive pulmonary damage with several large and small size tubercles distributed throughout the lung, together with progressive splenic and hepatic tissue destruction comparable to that observed in saline treated animals. However, animals immunized with rBCG85C showed a significantly reduced gross pathological damage, when compared to saline treated animals (*p*<0.05), as was evident from minimal involvement of the lungs with no evident sign of tissue damage in both liver and spleen (Fig. 2B).

### rBCG85C vaccination reduces granulomatous inflammation

**Figure 3:**
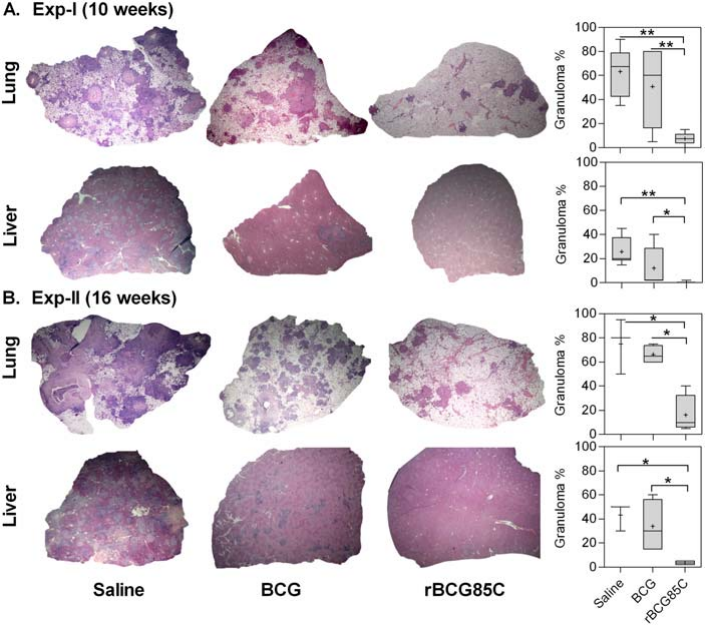
Histopathological changes in the lungs and liver of rBCG85C vaccinated animals following *M. tuberculosis* challenge. The figure depicts representative lower magnification photomicrographs of H&E stained, formalin fixed and paraffin embedded 5 µm sections of lung and liver of guinea pigs (n = 6) euthanized at (A) 10 weeks (Exp-I) and (B) 16 weeks (Exp-II) post-infection. Saline group is characterized by the presence of multiple coalescing granulomas with central necrotic core. rBCG85C immunized animals showed reduced granulomatous infiltration with only a few small and discrete granulomas in comparison to BCG vaccinated animals. Pulmonary and hepatic granuloma percent were measured and graphically represented by box plot, wherein median values are denoted by horizontal line, the mean is represented by ‘+’, inter quartile range by boxes, and the maximum and minimum values by whiskers. *, *p*<0.05 and **, *p*<0.01 (Mann-Whitney *U* test).

To evaluate the histopathological changes in the lungs and liver of immunized and saline treated animals, the tissue sections were stained with haematoxylin and eosin and granuloma percent was measured as described in the materials and methods. At 10 weeks post-infection, the type of lesions observed in the lungs of saline treated guinea pigs typically represented an advanced stage granuloma, with extensive coalescence of multiple granulomas covering 65% area of the lung sections (Fig. 3A). The lung granulomas in this group were characterized by extensive necrosis resulting in the loss of lung micro-architecture. The extent of granulomatous infiltration in BCG vaccinated animals was comparable to that observed in case of saline treated animals (Fig. 3A). However, the extent of necrosis in BCG vaccinated animals was relatively less, when compared to the saline treated animals. Immunization with rBCG85C resulted in a significant reduction in the granulomatous infiltration (8%), with the presence of very few small and discrete granulomas, when compared to the saline treated animals (*p*<0.01). The alveolar and bronchiolar structures in the surrounding areas were well preserved in this group. On comparing the pathological changes in liver (Fig. 3A), BCG immunized animals showed a relatively less granulomatous infiltration (12%), when compared to the saline treated animals (25%). However, vaccination with rBCG85C prevented hepatic damage as was evident from only a negligible (0–2%) granulomatous infiltration, when compared to BCG immunized animals (*p*<0.01).

At 16 weeks post-infection (Exp-II), the saline treated animals exhibited extensive granulomatous infiltration with coalescing necrotic granulomas (75%) effacing the pulmonary parenchyma (Fig. 3B). Granulomas observed in case of BCG immunized animals also represented a scenario similar to that observed in the saline treated animals showing 66% granulomatous infiltration. In contrast, immunization with rBCG85C preserved the pulmonary tissue organization with a significant reduction in pathological damage (16% granuloma, *p*<0.05) and necrosis. On comparing the pathological changes in liver (Fig. 3B), saline treated animals exhibited extensive granulomatous infiltration in the hepatic lobules, showing multiple coalescing foci of necrotic granulomas (43%). The reduction in hepatic damage in case of BCG immunization was variable, with percent granuloma ranging from 15–60%. Immunization with rBCG85C resulted in a significant reduction in hepatic damage with only a negligible granulomatous infiltration (3.5%, *p*<0.05).

### Reduction in pulmonary fibrosis with rBCG85C vaccination

**Figure 4:**
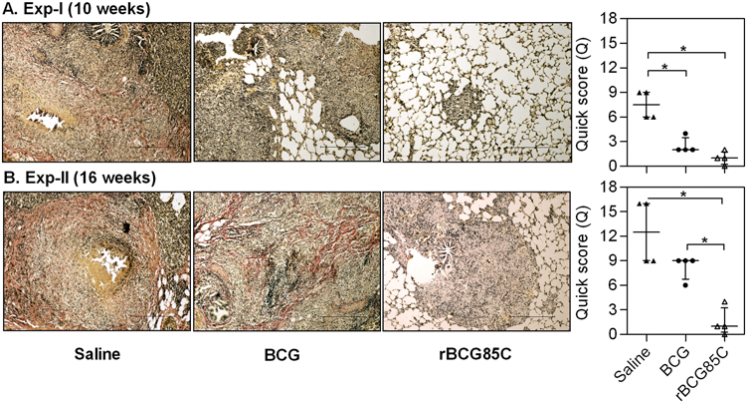
Influence of rBCG85C vaccination on pulmonary fibrosis. The representative photomicrographs of Van Gieson stained 5 µm lung sections of guinea pigs (n = 4) euthanized at (A) 10 weeks (Exp-I) and (B) 16 weeks (Exp-II) post-infection, depict significantly less collagen deposition (red color) in association with reduced granulomatous inflammation in rBCG85C immunized animals, when compared to BCG vaccinated group. Saline group is characterized by extensive fibrosis of granulomatous regions. Scale bar represents 1000 µm. Extent (Q) of pulmonary fibrosis was measured [Q = Intensity (I)×area (A) of staining] and represented graphically as median (±inter quartile range). *, *p*<0.05 (Mann-Whitney *U* test).

Development of necrosis and eventual fibrosis resulting in irreversible effacement of lung micro-architecture and respiratory failure are the primary features of progressive pulmonary TB in guinea pig model [33], [34]. Hence, in addition to the measurement of percent granuloma, the extent of collagen deposition was also determined. In case of saline treated animals at 10 weeks post-infection (Exp-I), extensive areas of collagen deposition in and around the granulomas were observed (Fig. 4A). Apart from the presence of collagen surrounding the necrotized core, irregular thick bands with a large number of foamy macrophages entrapped within the collagenous layer were also observed. However, a marked reduction in collagen deposition was observed in case of BCG immunized animals (*p*<0.05), wherein, the thin and diffused bands of collagen were primarily restricted to the periphery of granulomatous areas (Fig. 4A). Commensurate with the reduced granulomatous inflammation, lung sections derived from rBCG85C-immunized animals exhibited the presence of only a negligible amount of collagen. In Exp-II, paralleling the increase in granulomatous response observed at 16 weeks post-infection, a marked increase in collagen deposition was observed in the case of BCG as well as saline treated animals (Fig. 4B). However, rBCG85C immunized guinea pigs showed only a negligible collagen staining, when compared to both the control groups (*p*<0.05).

### Decline in the antigen load in pulmonary granulomas following rBCG85C vaccination

**Figure 5:**
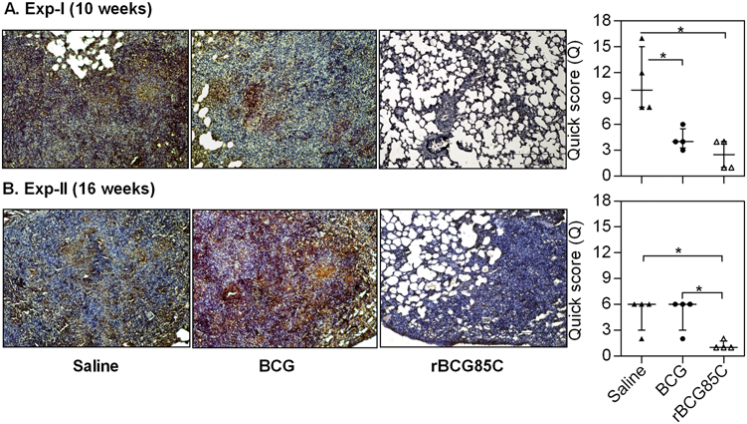
Reduced *M. tuberculosis* antigen load in pulmonary granulomas of rBCG85C vaccinated animals. The representative photomicrographs of 5 µm lung sections show immuno-histochemical staining (brown color) for Ag85 complex proteins in pulmonary granulomas at (A) 10 weeks (Exp-I) and (B) 16 weeks (Exp-II) post-infection. Immunization with rBCG85C in conjunction with reduced granulomatous inflammation showed a significantly lower antigen load at both 10 and 16 weeks post-infection, when compared to saline treated animals, which showed extensive staining surrounding and within the necrotic areas. BCG vaccination showed a significant reduction in antigen load at 10 weeks post-infection. At 16 weeks post-infection, extensive staining was observed in BCG immunized animals similar to that in case of saline-treated animals. Scale bar represents 1000 µm. Extent (Q) of staining was measured [Q = intensity (I)×area (A) of staining] and represented graphically as median (±inter quartile range). *, *p*<0.05 (Mann-Whitney *U* test).

To assess the effect of vaccination on clearance of antigen depots and/or bacillary remnants (a source of inflammation), IHC staining for *M. tuberculosis* antigens was carried out. Since, Ag85 complex proteins represent some of the predominant antigens localized both in the bacterial cell wall as well as in the secretory fraction, antibodies specific to Ag85 complex proteins were employed for *in situ* localization of these antigens as a marker for the presence of both live mycobacteria and mycobacterial remnants in the pulmonary granulomas. Immuno-localization provides advantage over acid fast staining, as the later requires the presence of intact cell wall of the bacilli, in contrast IHC can detect bacillary remnants, secreted antigens as well as the live bacilli. A reduced bacillary load and granulomatous inflammation in the lungs of rBCG85C-immunized animals suggested a commensurate clearance of antigenic depots from the pulmonary granulomas. On comparing the extent of antigen staining, extensive areas of intense staining within the granulomas were observed in the saline treated animals at 10 weeks post-infection (Fig. 5A). However, lung sections derived from animals vaccinated with BCG and rBCG85C showed a significantly reduced antigen load, when compared to the saline treated animals (*p*<0.05). On extending the time of euthanasia to 16 weeks, animals immunized with rBCG85C showed a significant reduction in antigen load in comparison to BCG immunized animals (*p*<0.05), with the latter exhibiting a staining pattern comparable to that observed in saline treated animals (Fig. 5B).

### Immuno-localization of IFN-γ and TNF-α in pulmonary granulomas

**Figure 6:**
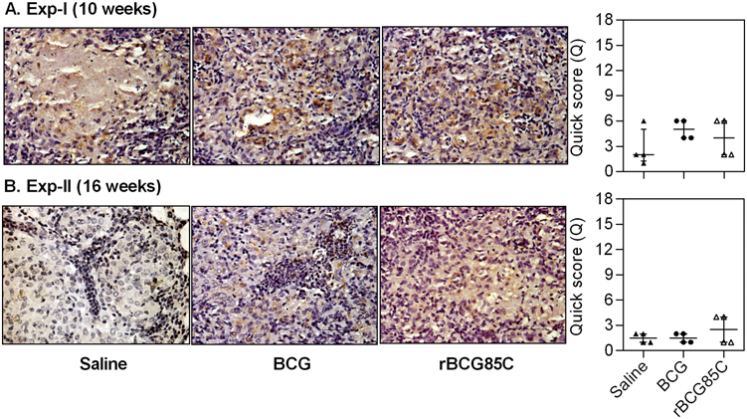
Immuno-localization of IFN-γ in pulmonary granulomas. The representative photomicrographs of 5 µm lung sections show immuno-histochemical staining (brown color) for IFN-γ in pulmonary granulomas at (A) 10 weeks (Exp-I) and (B) 16 weeks (Exp-II) post-infection. IFN-γ staining in both the vaccinated groups was found to be relatively higher at 10 weeks post-infection in comparison to the saline treated group. However, at 16 weeks post-infection, a comparable staining was observed in both the vaccinated and saline treated animals. Scale bar represents 200 µm. Extent (Q) of staining was measured [Q = Intensity (I)×area (A) of staining] and represented graphically as median (±inter quartile range). (Mann-Whitney *U* test).

**Figure 7:**
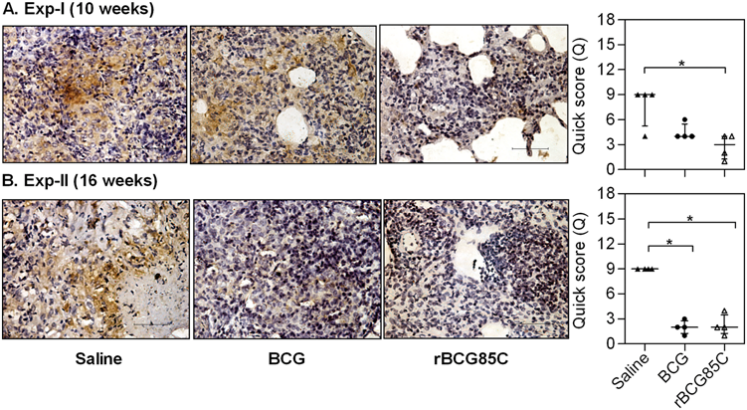
Immuno-localization of TNF-α in pulmonary granulomas. The representative photomicrographs of 5 µm lung sections show immuno-histochemical staining (brown color) for TNF-α in pulmonary granulomas at (A) 10 weeks (Exp-I) and (B) 16 weeks (Exp-II) post-infection. Immunization with rBCG85C in conjunction with reduced granulomatous inflammation showed a significantly reduced staining for TNF-α at both 10 and 16 weeks post-infection, when compared to saline treated animals, which showed extensive staining surrounding and within the necrotic areas. The extent of staining in BCG group was relatively higher at 10 weeks in comparison to rBCG85C, however, at 16 weeks a comparable staining was observed. Scale bar represents 200 µm. Extent (Q) of staining was measured [Q = intensity (I)×area (A) of staining] and represented graphically as median (±inter quartile range). *, *p*<0.05 (Mann-Whitney *U* test).

Immuno-histochemical staining of the lung tissues showed the presence of IFN-γ and TNF-α in all the groups and was marked by their abundance in the granulomatous lesions compared to that in the non-granulomatous areas. However, the extent of staining varied among the groups. In Exp-I, based on the area and intensity of staining, the extent of IFN-γ expression in granulomatous regions indicated a comparable presence of this cytokine in the lungs of BCG and rBCG85C immunized animals at 10 weeks post-infection (Fig. 6A). Majority of the saline treated animals showed relatively very low levels of this cytokine localized primarily in the areas infiltrated with macrophages. Lung sections from BCG immunized animals showed moderate levels of IFN-γ staining in the areas infiltrated by both macrophages and lymphocytes. A similar pattern of IFN-γ staining was also observed in case of rBCG85C-immunized animals (Fig. 6A). At this time point, a very high level of TNF-α was observed in case of saline treated animals (Fig. 7A). TNF-α was localized extensively in the necrotic areas as well as in the macrophages and extra cellular spaces within the advanced coalescent granulomas. Immunization with BCG resulted in the reduced amounts of TNF-α expression primarily localized inside the macrophages and in non-necrotic granulomatous areas. In contrast, rBCG85C-vaccinated animals showed only a negligible staining for TNF-α with its presence restricted primarily to the granuloma core (Fig. 7A). On extending the time of euthanasia to 16 weeks in Exp-II, an overall reduced IFN-γ levels were observed in all the groups with no significant differences (Fig. 6B). As observed at 10 weeks, at this time point also, the levels of TNF-α were found to be significantly lower in case of both the vaccinated groups, when compared to the saline treated animals (*p*<0.05) (Fig. 7B)

### Modulation of host gene expression in the lungs by rBCG85C vaccination

**Figure 8:**
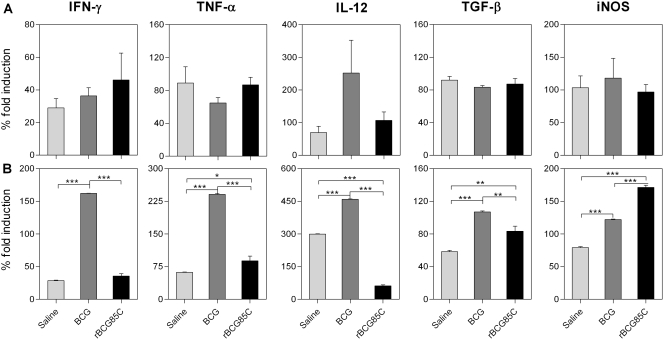
Modulation of host gene expression in the lung by rBCG85C vaccination. Expression of various cytokines and iNOS was measured in the lung tissues of vaccinated and saline treated guinea pigs (n = 3) at (A) 10 weeks (Exp-I) and (B) 16 weeks (Exp-II) post-infection by semi-quantitative real time RT-PCR using gene specific primers (Table S2). The data were normalized to 18S rRNA levels and then normalized to the values of uninfected animals to obtain ΔΔCt values. The % fold induction values were measured (2 ^−ΔΔCt^×100) and are graphically represented as mean (±SE). Immunization with rBCG85C resulted in relatively lower levels of pro-inflammatory cytokines IL-12, IFN-γ and TNF-α and moderate level of anti-inflammatory cytokine TGF-β with a significant up-regulation of iNOS expression, when compared to both BCG immunized and saline treated animals. *, *p*<0.05; **, *p*<0.01 and ***, *p*<0.001 (One way ANOVA).

Profiling of various cytokines and iNOS by real time RT-PCR using gene specific primers (Table S2) and RNA isolated from the lungs of vaccinated and non-vaccinated guinea pigs revealed a distinct pattern of IFN-γ, TNF-α, TGF-β, IL-12 and iNOS expression at both 10 weeks and 16 weeks post *M. tuberculosis* infection. The data for real time PCR is graphically shown in Fig. 8 as % fold induction in the levels of different cytokines and iNOS relative to the induction in the uninfected (and non-vaccinated) animals. The relative proportion of IFN-γ and TNF-α in the transcript pool obtained from the whole lung homogenate was found to be in striking contrast to the results obtained from immuno-localization of these cytokines by IHC. At 10 weeks post-infection (Exp-I), both vaccinated and saline treated guinea pigs showed a comparable fold induction of these cytokines at mRNA level (Fig. 8A). However, at 16 weeks post-infection (Exp-II), a significant up-regulation of both the inflammatory cytokines (IFN-γ and TNF-α) was observed in BCG immunized guinea pigs (Fig. 8B), when compared to the saline treated animals (*p*<0.001). In case of rBCG85C-immunized animals, a significant reduction in the bacillary load and pathological damage was accompanied by a marked reduction in the inflammatory responses as was evident from the reduced transcript levels of these cytokines, when compared to BCG immunized animals (*p*<0.001). Although, rBCG85C immunized animals showed marginally higher levels of TNF-α in comparison to the saline treated animals, the IFN-γ levels in both the groups remained comparable (Fig. 8B). Surprisingly, in the saline group, despite a very high bacillary load and extensive pulmonary tissue damage, very low levels of IFN-γ and TNF-α were observed at both the time points, which was in striking contrast to relatively very high levels of TNF-α staining observed in pulmonary granulomas in this group by IHC.

In addition to these two major cytokines (IFN-γ and TNF-α) that have been implicated in TB associated pathology, the combinatorial effect of IL-12p40 (pro-inflammatory) and TGF-β. (anti-inflammatory) on granuloma formation, tissue remodeling and disease resolution was also studied by measuring the expression levels of these cytokines. At 10 weeks following *M. tuberculosis* infection, BCG immunized guinea pigs showed relatively higher levels of IL-12p40, when compared to saline treated animals (Fig. 8A). Immunization with rBCG85C resulted in only a marginal augmentation in the expression levels of IL-12p40 over saline treatment. With progression of disease to 16 weeks (Fig. 8B), a marked shift in the expression profile of this cytokine was observed, wherein, rBCG85C immunized animals showed a considerable reduction in the levels of IL-12p40, when compared to saline treated animals (*p*<0.001). However, there was a significant up-regulation of this cytokine in BCG immunized guinea pigs, when compared to the saline group (*p*<0.001).

On comparing the expression levels of TGF-β, at 10 weeks post-infection (Fig. 8A), no considerable differences were observed in the vaccinated and saline treated animals. However, at 16 weeks post-infection (Fig. 8B), both BCG and rBCG85C immunized animals showed increased levels of TGF-β, when compared to the saline treated animals. The extent of up-regulation of TGF-β in BCG group was significantly higher in comparison to rBCG85C-immunized animals (p<0.01).

Production of highly reactive nitric oxide intermediates (RNI) represents one of the key mycobactericidal mechanisms employed by the activated macrophages and iNOS is the principal enzyme involved in the generation of RNI by phagocytes [35], [36]. Hence, in addition to studying the effect of vaccination on induction of various cytokines, expression of iNOS was also measured. On comparing the transcription profile of iNOS in lungs obtained from various vaccination groups, at 10 weeks post-infection, despite the significant differences in bacillary load and pathological damage, no significant differences in the levels of iNOS were observed between the vaccinated and non-vaccinated animals (Fig. 8A). However, at 16 weeks, distinct differences in the levels of iNOS expression were observed among various groups (Fig. 8B). Immunization with both BCG and rBCG85C resulted in a marked elevation in iNOS levels in comparison to the saline treatment (*p*<0.001). However, the extent of up-regulation was significantly higher in rBCG85C group in comparison to BCG immunized animals (*p*<0.001), which corroborate well with the differences in the bacillary load observed among these groups at this time point.

## Discussion

This study demonstrates a significant enhancement in the protective efficacy of BCG by over expression of Ag85C- an immuno-dominant antigen of *M. tuberculosis* and correlation of this superior protection with the immuno-pathological events attributable to modulation of the cytokine profile of pulmonary granulomas. The parameters used for the evaluation of protective efficacy following an aerosol challenge with *M. tuberculosis* were, (i) bacillary load in lung and spleen and (ii) pathological changes in lung, liver and spleen. The challenge dose employed in this study resulted in an early manifestation of severe disease symptoms and thus allowed discrimination between the protective efficacies of rBCG and parent BCG vaccines within a reasonable time frame. At 10 weeks post-infection, vaccination with rBCG85C resulted in a significantly reduced bacillary load in the lungs (∼87 folds) along with a marked reduction in hematogenous spread to the spleen (∼360 folds) in comparison to vaccination with the parental BCG strain. This reduced bacillary load was also accompanied by a marked reduction in the pulmonary, splenic and hepatic pathology. On extending the interval between vaccination and challenge (to 12 weeks) and between challenge and euthanasia (to 16 weeks), rBCG85C continued to impart a relatively superior protection with a remarkably greater control on bacillary multiplication in the lungs (∼9 folds) and a successful restriction of the hematogenous spread of tubercle bacilli to spleen (∼100 folds) in comparison to immunization with the parent BCG strain. The importance of these variables was clearly emphasized in a comprehensive study carried out by EU TB Vaccine Cluster, wherein, the superiority of many candidate vaccines over BCG was demonstrated by employing a high dose of challenge with an extended post challenge evaluation period [37].

Being a localized infection, the control of pulmonary TB largely depends on the orchestration of various cellular components of the immune system and a coordinated interplay of various pro- and anti-inflammatory cytokines at the foci of infection [38], [39]. Several studies have suggested that often it requires more than a single cytokine to influence the cell mediated tissue damage in response to microbial infection and a fine-tuning of multiple cytokines is *de rigueur* for an effective clearance of the pathogen [39], [40]. In the absence of vaccination, the clinical manifestation of progressive end-stage TB in guinea pigs is known to be associated with a strong inflammatory response to the persistent antigens or bacilli leading to extensive necrosis and progressive fibrosis [33]. However, an efficient vaccine is expected to prime the immune system to generate an efficiently regulated and targeted response for an effective microbial and antigenic clearance, minimizing the collateral damage to the host. Immuno-localization of Ag85 complex proteins–some of the most abundant proteins of *M. tuberculosis*, as a marker of the mycobacterial antigen load, showed elevated levels of these antigens in the granulomas as observed in case of saline treated animals. This increased antigen load was found to be associated with the production of superfluous amount of TNF-α, unwarranted inflammation, tissue destruction and excessive collagen deposition. However, in addition to the bacillary clearance, rBCG85C mediated immune responses resulted in reduced antigen load indicating an effective removal of mycobacterial antigens and/or the bacillary remnants. A corresponding reduction in the extent of granulomatous inflammation and fibrosis in this group further substantiated the fact that an effective removal of the residual antigenic depots from the sites of infection is essential for the resolution of granulomatous lesions. More over, reduction in the levels of IFN-γ and TNF-α, towards the later stage of disease in case of the rBCG85C-immunized animals, further signifies the fact that, although, induction of these cytokines following *M. tuberculosis* infection is known to be essential for the initial containment of the bacilli [39], [41], a subsequent reduction in the levels of these cytokines is crucial for the resolution of granulomatous lesions, as observed in this study. However, an apparent lack of concordance in the levels of these cytokines (as measured by IHC) with the antigen load and granulomatous inflammation in the lungs of BCG immunized animals suggests involvement of additional cytokines and cellular components in the pulmonary inflammation and tissue damage not measured in this study.

Comparison of mRNA expression profile for various cytokines showed that at 10 weeks post-infection, no specific pattern was evident for any vaccine regimen. However, at 16 weeks a characteristic cytokine profile was found to be associated with the history of vaccination. BCG immunization caused up-regulation of both the pro-inflammatory cytokines (IL-12, IFN-γ and TNF-α) as well as anti-inflammatory cytokine (TGF-β). These counter-acting mechanisms could neither reduce the exaggerated granulomatous response, nor control excessive bacillary multiplication. In contrast, rBCG85C immunization resulted in significantly lowered levels of IL-12, IFN-γ and TNF-α and a marginal decline in the levels of TGF-β in comparison to BCG vaccination. The reduction in the levels of the pro-inflammatory cytokines has been reported to be essential for the resolution of granulomatous inflammation and thus crucial for alleviation of disease symptoms (reviewed in [42]). Besides, the up-regulation of iNOS in case of rBCG85C vaccination towards the later stage of the disease, possibly resulted in an efficient killing of the bacilli, in comparison to the BCG vaccinated animals. Such mycobactericidal effect of iNOS although has been reported in several *in vitro* and *in vivo* studies [36], [43], [44], its influence in the context of vaccine response in guinea pig model for such a prolonged period has been investigated for the first time in this study.

In all TB vaccine related studies, BCG has been used as the gold standard to pronounce the worthiness of a new vaccine candidate, because it is the failure of BCG in the adult human population that has necessitated the development of a new TB vaccine in the first place. However, this convention suffers from a caveat–a new vaccine is required for protection in humans, wherein, BCG does not work well; on the other hand, a new vaccine cannot progress to human trials without proving its superiority to BCG in animal models in which BCG works rather efficiently. Hence, it has been difficult to develop vaccines, which would ensure a superior protection over BCG in animal models. It is thus not surprising that in spite of a large number of vaccine related studies, merely 9 vaccine regimens have progressed to various stages of human clinical trials (reviewed in [45]). These vaccines have shown a better or equal performance in comparison to BCG in their ability (i) to reduce the bacillary load in lung and spleen and/or (ii) to reduce pathological damage and/or (iii) to perform better in time to death assay. While survival assay represents the most dependable tool to evaluate the protective efficacy of TB vaccines in animal models, due to infrastructure and time constraints involved in these long drawn studies, it has been customary to first evaluate TB vaccines in time-bound studies and then channelize the promising ones through survival assays [37], [46]. We have not yet carried out the survival assays with the rBCG85C vaccine however, the 16 weeks assay carried out to evaluate its protective efficacy in a highly relevant guinea pig model of TB shows that at least on the basis of such evaluations and their comparison with all the vaccines that have already progressed to clinical trials, rBCG85C imparts a remarkable protection. The enduring protection observed in this study gives enough reasons to postulate that if an open-ended study is carried out, rBCG85C vaccine in all likelihood would show enhanced survival of guinea pigs.

Furthermore, we have attempted to provide an insight into the association between the protective efficacy imparted by an efficient vaccine and the cytokine milieu in the pulmonary granuloma in guinea pigs for such a prolonged post-challenge period. Such a comprehensive evaluation of the temporal and spatial variations in immune components, if carried out along with the long-term survival assays, may help in removing the existing paradox associated with the role of various cellular components and cytokines in mediating protection against *M. tuberculosis* infection. Our future efforts would focus on these aspects.

## Supporting Information

Table S1 Post-mortem gross pathological scoring system. The table illustrates the gross pathological scoring system used for visual scoring of lesions in lung, liver and spleen of guinea pigs infected with *M. tuberculosis*. Mitchison's virulence scoring system was modified and equal emphasis was given to every organ and scores were graded as 1–4.
(0.04 MB DOC)

Table S2 Primer sequences used for real time PCR.
(0.03 MB DOC)
