# Supplementary material for: Enhanced and Enduring Protection against Tuberculosis by Recombinant BCG-Ag85C and Its Association with Modulation of Cytokine Profile in Lung
Source: PLoS One. 2008 Dec 4;3(12):e3869. doi: 10.1371/journal.pone.0003869 (PMC2586085; doi:10.1371/journal.pone.0003869)
Supplement: Table S1 — Post-mortem gross pathological scoring system. The table illustrates the gross pathological scoring system used for visual scoring of lesions in lung, liver and spleen of guinea pigs infected with M. tuberculosis. Mitchison's virulence scoring system was modified and equal emphasis was given to every organ and scores were graded as 1–4. (0.04 MB DOC) [file pone.0003869.s001.doc]

**SUPPORTING INFORMATION**

#### Table S1. Post-mortem gross pathological scoring system.

The table illustrates the gross pathological scoring system used for visual scoring of lesions in lung, liver and spleen of guinea pigs infected with *M. tuberculosis*. Mitchison’s virulence scoring system was modified and equal emphasis was given to every organ and scores were graded as 1-4.

#### Table S1. Post-mortem gross pathological scoring system

| **Characteristics** | **Mitchison’s scores** | | **Scores used^a^** |
| --- | --- | --- | --- |
| Lung | | | |
| Heavy involvement with numerous large tubercles | | 20 | 4 |
| Moderate involvement with occasional large tubercles or numerous small tubercles | | 15 | 3 |
| Scanty involvement with up to 4 large tubercles or moderate number of small tubercles | | 10 | 2 |
| Minimal involvement with scanty small tubercles | | 5 | 1 |
| Liver | | | |
| Heavy involvement with numerous large tubercles and some areas of necrosis | | 30 | 4 |
| Moderate involvement with moderate number of large tubercles or numerous small tubercles, no area of necrosis | | 23 | 3 |
| Scanty involvement with scanty tubercles, easily visible | | 15 | 2 |
| Minimal involvement with just visible tubercles or no tubercles | | 8 | 1 |
| Spleen | | | |
| Heavy involvement with numerous large tubercles and areas of necrosis | | 40 | 4 |
| Moderate involvement with numerous large tubercles but a few areas of necrosis or markedly enlarged spleen with numerous small tubercles | | 30 | 3 |
| Scanty involvement with a few large tubercles or numerous small but easily visible tubercles | | 20 | 2 |
| Minimal involvement with a few small visible tubercles or no tubercle | | 10 | 1 |
| ^a^ Mitchison’s virulence scoring system [29] was modified and equal weight was given to every organ. Based on the extent of involvement, number and size of tubercles, areas of inflammation and necrosis, gross pathological scores were graded from1-4. | | | |
