# Supplementary material for: Enhanced and Enduring Protection against Tuberculosis by Recombinant BCG-Ag85C and Its Association with Modulation of Cytokine Profile in Lung
Source: PLoS One. 2008 Dec 4;3(12):e3869. doi: 10.1371/journal.pone.0003869 (PMC2586085; doi:10.1371/journal.pone.0003869)
Supplement: Table S2 — Primer sequences used for real time PCR. (0.03 MB DOC) [file pone.0003869.s002.doc]

Table S2. Primer sequences used for real time PCR

| Gene Name^a^ (Accession No.) | Primer sequence ^b^ |
| --- | --- |
| IFN-γ  (AY151287) | F, 5’ aca agg tgc agg ctt tca aaa 3’  R, 5’ ttg gcg ctg gac atg ct 3’ |
| TNF-α  (U77036) | F, 5’ tgg ccc ccc ctt cag a 3’  R, 5’ tgt cat tat cgt ttt gag aag ctg at 3’ |
| TGF-β (AF191297) | F, 5’ tgt gtg cgg cag ctc tac at 3’  R, 5’ agt tgg cat ggt agc cct tg 3’ |
| IL-12 (AB025724) | F, 5’ ggt ggt ggt gga tgc tat tc 3’  R, 5’ cag gtt ctt cgg tgg gtc t 3’ |
| iNOS^c^ | F, 5’ gca gca gcg gct tca ca 3’  R, 5’ aca tcc aaa cag gag cgt cat t 3’ |
| 18S^d^ | F, 5’ tgc atg gcc gtt ctt agt tg 3’  R, 5’ agt tag cat gcc aga gtc tcg tt 3’ |
| ^a^ The primer sequences were designed by using cDNA sequences as described in materials and methods.  ^b^ F, forward; R, reverse  ^c^ ,^d^ The primer sequences used were as described earlier [31,32]. | |
